# Haploidentical and matched unrelated donor allogeneic hematopoietic stem cell transplantation offer similar survival outcomes for acute leukemia

**DOI:** 10.1002/cnr2.2060

**Published:** 2024-04-10

**Authors:** Yin‐Che Wang, Cheng‐Lun Lai, Tsung‐Chih Chen, Jyh‐Pyng Gau, Chieh‐Lin Jerry Teng

**Affiliations:** ^1^ Division of Hematology/Medical Oncology, Department of Medicine Taichung Veterans General Hospital Taichung Taiwan; ^2^ Department of Post‐Baccalaureate Medicine, College of Medicine National Chung Hsing University Taichung Taiwan; ^3^ Division of Hematology and Oncology, Department of Medicine Taipei Medical University Hospital; ^4^ Ph.D. Program in Translational Medicine National Chung Hsing University Taichung Taiwan; ^5^ Rong Hsing Research Center for Translational Medicine National Chung Hsing University Taichung Taiwan; ^6^ School of Medicine Chung Shan Medical University Taichung Taiwan; ^7^ Department of Life Science Tunghai University Taichung Taiwan

**Keywords:** hematopoietic stem cell transplantation, human leukocyte antigen, leukemia, survival analysis

## Abstract

**Background:**

Haploidentical hematopoietic stem cell transplantation (haplo‐HSCT) has emerged as an effective approach for acute leukemia, primarily due to the inherent difficulty in finding human leukocyte antigen‐matched unrelated donors (MUD). Nevertheless, it remains uncertain whether haplo‐HSCT and MUD‐HSCT can provide comparable outcomes in patients with acute leukemia.

**Aims:**

This study aimed to assess the overall survival (OS) and leukemia‐free survival (LFS) outcomes between the MUD‐HSCT and haplo‐HSCT groups.

**Methods and results:**

This retrospective analysis encompassed adult patients with acute leukemia undergoing the initial allo‐HSCT. Among these 85 patients, we stratified 33 patients into the MUD‐HSCT group and 52 to the haplo‐HSCT group. The primary outcomes were OS and LFS. The median OS was not reached in the haplo‐HSCT group, while it reached 29.8 months in patients undergoing MUD‐HSCT (*p* = .211). Likewise, the median LFS periods were 52.6 months in the haplo‐HSCT group and 12.7 months in the MUD‐HSCT group (*p* = .212). Importantly, neither the OS nor LFS showed substantial differences between the MUD‐HSCT and haplo‐HSCT groups. Furthermore, univariate analyses revealed that haplo‐HSCT did not demonstrate a significantly higher risk of worse LFS (hazard ratio [HR], 0.69; 95% confidence interval [CI], 0.38–1.25; *p* = .216) or OS (HR, 0.67; 95% CI, 0.36–1.26; *p* = .214) than MUD‐HSCT. Notably, a high European Group for Blood and Marrow Transplantation risk score (HR, 1.44; 95% CI, 1.10–1.87; *p* = .007) and non‐complete remission (HR, 2.48; 95% CI, 1.17–5.23; *p* = .017) were significantly correlated with worse OS.

**Conclusion:**

Haplo‐HSCT may serve as an alternative to MUD‐HSCT for the treatment of acute leukemia, offering similar survival outcomes.

## 1 INTRODUCTION

Allogeneic hematopoietic stem cell transplantation (allo‐HSCT) stands as one cornerstone among post‐remission therapies and holds the potential to provide a curative treatment option for individuals with acute myeloid leukemia (AML)^1^, ^2^ and acute lymphoblastic leukemia (ALL).^3^ Typically, the preference in allo‐HSCT leans towards utilizing hematopoietic stem cells from a human leukocyte antigen (HLA)‐matched sibling donor (MSD) because of the comparatively lower chances of graft‐versus‐host disease (GVHD) and transplant‐related mortality.^4^ However, the accessibility to an HLA‐MSD is frequently constrained, with more than 70% of patients lacking a suitable donor.^5^ When an HLA‐MSD donor is not available, HLA‐matched unrelated donors (MUD) are often pursued as a substitute donor option for allogeneic hematopoietic stem cells.

In recent years, haploidentical HSCT (haplo‐HSCT) has gained popularity as a viable option because finding a suitable HLA‐MUD can be challenging. Haploidentical donors provide a readily accessible source of allogeneic hematopoietic stem cells for nearly all individuals requiring allo‐HSCT. However, haplo‐HSCT has several limitations. For example, haplo‐HSCT is known to elevate the incidence of cytomegalovirus (CMV) infection^6^ and GVHD,^7^ which consequently leads to suboptimal overall survival (OS) compared with MSD‐HSCT and MUD‐HSCT.^8^ More specifically, while MSD‐HSCT remains the preferred option for AML in the first complete remission (CR), outcomes of haplo‐HSCT are comparable to those of MSD‐HSCT in AML patients with high cytogenetic risk.^9^ Nevertheless, there have been notable advancements in the outcomes of haplo‐HSCT in recent years. These improvements can be attributed to the implementation of effective CMV prophylactic strategies^10^, ^11^ and the utilization of post‐transplantation cyclophosphamide (PTCY) treatment to mitigate GVHD.^12^, ^13^ Currently, emerging evidence suggests that haplo‐HSCT can yield outcomes comparable to those of MUD‐HSCT for various hematological malignancies.^14^, ^15^

The impact of these results on patients in Asian countries is uncertain because of potential variations in the genetic diversity of MUDs across different regions. Additionally, Asia is considered a CMV‐endemic area,^16^ and therefore, more severe CMV reactivation may occur in haplo‐HSCT recipients in Asia than in other regions. Taking these factors into account, we initiated a retrospective study in Taiwan to explore the feasibility of haplo‐HSCT as a substitute for MUD‐HSCT in the acute leukemia treatment.

The primary study objective of the current investigation was to perform a comparative analysis of OS and leukemia‐free survival (LFS) in individuals of acute leukemia receiving either haplo‐HCT or MUD‐HSCT. Additionally, we evaluated engraftment times and the chance of both acute and chronic GVHD in these two patient cohorts.

## 2 METHODS AND MATERIALS

### 2.1 Patients

We performed a retrospective analysis of medical files of 85 consecutive adult patients undergoing their initial MUD‐HSCT (*n* = 33) or haplo‐HSCT (*n* = 52) at Taichung Veterans General Hospital between January 2010 and December 2021 for AML (*n* = 56), ALL (*n* = 27), or mixed‐phenotype acute leukemia (*n* = 2). To initiate diagnostic processes, every patient was subjected to bone marrow aspiration and biopsy. The confirmation of leukemia diagnosis relies on an integrated approach involving flow cytometry, cytogenetic analysis, and molecular assays, basically aligning with the World Health Organization Classification of Tumors of Hematopoietic and Lymphoid Tissues, 2017. Molecular assays specifically included routine testing for FLT3 ITD/TKD and NPM1 mutations. For a select group of patients, next‐generation sequencing was also employed. We utilized the peripheral blood as the only source of hematopoietic stem cells. The HLA typing was conducted using high‐resolution DNA‐based methods, specifically at the allele level. Recipients of matched unrelated donors were matched at the allele‐level at HLA‐A, ‐B, ‐C, ‐DQ and ‐DR. Haploidentical donors were found to have mismatches at two or more HLA loci. The median duration of follow‐up was 20.8 months, with 20.7 months for the haplo‐HSCT group and 26.7 months for the MUD‐HSCT group (*p* = .379). The last follow‐up date recorded was December 31, 2022. The Institutional Review Board of Taichung Veterans General Hospital approved the study (CE23259C) and waived the requirement of informed consent because of the retrospective study nature.

### 2.2 Definitions and outcome measurement

The primary analytic endpoints encompassed OS and LFS. OS was defined as the time elapsed from the commencement of allo‐HSCT until the event of death due to any causes. LFS, on the other hand, was delineated as the duration from the commencement of allo‐HSCT to the identification of leukemia recurrence, substantiated by pathological evidence, or death from any causes. Additionally, we conducted a comparative analysis of the engraftment timeline and the occurrence chances of acute and chronic GVHD between MUD‐HSCT and haplo‐HSCT groups. The immunological recovery was defined as attaining a CD4 count of ≥200 cells/mm^3^. The initial disease risk for AML^17^ and ALL,^18^ Charlson comorbidity index,^19^ disease status before transplantation, risk score of European Group for Blood and Marrow Transplantation (EBMT),^20^ and disease risk index^21^ were defined accordingly. Severe infections during the transplantation process were characterized as occurrences of bacteremia or fungemia throughout the course of HSCT.

### 2.3 Conditioning regimens

The non‐myeloablative regimen conditioning to the MUD‐HSCT group comprised total body irradiation (TBI) at a dosage of 200 cGy on Day 7, fludarabine at 30 mg/m^2^/day from Day 6 to Day 2, and cyclophosphamide at a dosage of 10 mg/kg/day from Day 5 to Day 2. In terms of myeloablative conditioning regimens for the MUD‐HSCT group, patients diagnosed with AML typically underwent BuCy2 (Busulfan: 2.4 mg/kg/day on Days 7 and 4; cyclophosphamide: 60 mg/kg/day on Days 3 and 2) therapy. The ALL patients were conditioned by a TBI‐based regimen, which included a total dose of 1200 cGy, in conjunction with cyclophosphamide (60 mg/kg/day administered on Days 3 and 2). For patients undergoing haplo‐HSCT, we adapted the John Hopkins protocol for both non‐myeloablative^12^ and myeloablative^22^ conditioning regimens.

### 2.4 GVHD prophylaxis

In the current study, cyclosporine, mycophenolic acid, and anti‐thymocyte globulin (ATG) from a rabbit source served as the primary immunosuppressants for GVHD prophylaxis across both groups of patients. The cyclosporine trough levels were targeted to be within the range of 150–250 ng/mL for all participants. In the MUD‐HSCT group, ATG was administered at a dosage of 2 mg/kg/day from Day 4 to Day 2, with cyclosporine treatment commencing on Day 2. Starting from Day 2, mycophenolic acid was given at a dosage of 720 mg twice daily.

For the haplo‐HSCT group, ATG was administered at the same dosage of 2 mg/kg/day but from Day 3 to Day 2. Post‐transplant cyclophosphamide (PTCy), at a dose of 50 mg/kg/day IV, was given on Days 3 and 4. The administration of cyclosporine and mycophenolic acid began on day 5. For all study participants, the cyclosporine dose was gradually reduced starting from day 90, and a phased discontinuation of mycophenolic acid commenced from day 60.

### 2.5 Statistical analysis

Categorical variables were compared using the chi‐squared test, while continuous variables were analyzed using the independent *t* test. To compute OS and LFS, the Kaplan–Meier method was utilized, and the log‐rank test was used to assess distinctions between MUD‐HSCT and haplo‐HSCT groups. Factors associated with LFS and OS were identified using logistic regression and Cox proportional hazards models. The results are presented as hazard ratios (HR) accompanied by the corresponding 95% confidence intervals (CI). Statistical significance was determined as *p* < .05. All statistical analyses were conducted using SPSS for Windows, version 26.0.

## 3 RESULTS

### 3.1 The comparison of patient characteristics

We observed comparable sex distribution (*p* = .971), median age at the time diagnosis (*p* = .951), Charlson comorbidity index (*p* = .520), and pre‐HSCT disease status (*p* = .601) in both groups. Nonetheless, it is noteworthy that the MUD‐HSCT group exhibited a greater percentage of individuals with high‐risk diseases when comparing to the haplo‐HSCT group (39.4% vs. 30.8%; *p* = .042). Moreover, a larger percentage of patients in the haplo‐HSCT group underwent non‐myeloablative conditioning regimens compared to those in the MUD‐HSCT group (88.5% vs. 24.2%; *p* < .001). No statistically substantial differences were noted between the two groups in terms of donor sex (*p* = .805), donor age (*p* = .078), ABO incompatibility (*p* = .116), and CD34+ cell count (*p* = .290) (Table 1).

**TABLE 1:** Patient characteristics (*n* = 85).

Variable	Haplo‐HSCT (*n* = 52)		MUD‐HSCT (*n* = 33)		*p*
Sex					.971
Male (*n*, %)	25	48.1%	16	48.5%	
Female (*n*, %)	27	51.9%	17	51.5%	
Age at leukemia diagnosis (years, median range)	47.5	18–74	46	20–70	.951
Type of leukemia					.130
AML (*n*, %)	37	71.2%	19	65.9%	
ALL (*n*, %)	15	28.8%	12	31.8%	
MPAL (*n*, %)	0	0%	2	2.4%	
Initial disease risk					.042
High (*n*, %)	16	30.8%	13	39.4%	
Low/Intermediate (*n*, %)	33	63.5%	13	39.4%	
Unknown (*n*, %)	3	5.8%	7	21.2%	
Charlson comorbidity index (median, range)	2	2–9	2	2–6	.520
CMV IgG (+) in recipients (*n*, %)	40	76.9	21	63.6%	.185
Pre‐HSCT status					.601
CR1 (*n*, %)	35	67.3%	22	66.7%	
CR2 (*n*, %)	11	21.2%	5	15.2%	
Non‐CR (*n*, %)	6	11.5%	6	18.2%	
Follow‐up time (median, range; months)	20.7	1.1–94.7	26.7	0.8–151	.379
Time from diagnosis to HSCT (median, range; days)	186	95–1290	169	82–1119	.760
EBMT risk score (median, range)	3	1–6	3	1–6	.380
Disease risk index					.357
Low/Intermediate (*n*, %)	38	73.1%	21	63.6%	
High/very high (*n*, %)	14	26.9%	12	36.4%	
Condition protocol					<.001
Myeloablative (*n*, %)	6	11.5%	25	75.8%	
Non‐myeloablative (*n*, %)	46	88.5%	8	24.2%	
Donor sex					.805
Male (*n*, %)	36	69.2%	22	66.7%	
Female (*n*, %)	16	30.8%	11	33.3%	
Age of the donor (years, median, range)	31	9–61	36	23–53	.078
CMV IgG (+) donors (*n*, %)	35	67.3%	25	75.8%	.405
ABO incompatibility (n, %)	24	46.2%	21	63.6%	.116
CD34+ cell count (median, range; ×10^6^/kg)	6.75	3.21–18.63	5.63	2.44–22.41	.290

Abbreviations: ALL, acute lymphocytic leukemia; AML, acute myeloid leukemia; CMV, cytomegalovirus; CR, complete remission; EBMT, European Group for Blood and Marrow Transplantation; Haplo‐HSCT, haploidentical hematopoietic stem cell transplantation; MPAL, mixed‐phenotype acute leukemia; MUD‐HSCT, matched unrelated donor hematopoietic stem cell transplantation.

### 3.2 Outcome comparisons during allo‐HSCT

The patients in the haplo‐HSCT group and MUD‐HSCT group exhibited comparable engraftment rates (100% vs. 97%, *p* = .207). The median engraftment days for these two groups were 16.2 ± 3.9 and 13.9 ± 9.3 days (*p* = .119). Individuals in the haplo‐HSCT group experienced a substantially prolonged duration of hospitalization when compared to those in the MUD‐HSCT group (41.0 ± 18.2 vs. 31.9 ± 15.1 days; *p* = .018). Importantly, these two groups of patients had comparable incidences of bacteremia and fungemia during allo‐HSCT (9.6% vs. 12.1%; *p* = .730; Table 2).

**TABLE 2:** Outcome comparison.

Variable	Haplo‐HSCT (*n* = 52)		MUD‐HSCT (*n* = 33)		*p*
Engraftment (*n*, %)	52	100%	32	97%	.207
Engraftment time (days, median ± SD)	16.2 ± 3.9		13.9 ± 9.3		.119
Length of hospital stay for HSCT (mean ± SD)	41.0 ± 18.2		31.9 ± 15.1		.018
Bacteremia and fungemia during HSCT (*n*, %)	5	9.6%	4	12.1%	.730
Overall survival (months, median)	Not‐reached		29.8		.211
90‐day survival (*n*, %)	49	94.20%	29	87.90%	.298
1‐year survival (*n*, %)	38	73.10%	21	63.60%	.339
Leukemia‐free survival (months, median)	52.6		12.7		.212
Ninety‐day‐survival (*n*, %)	46	88.5%	26	78.8%	.236
One‐year‐survival (*n*, %)	35	67.3%	18	54.5%	.212
Incidence of acute GVHD (any grade) (*n*, %)	30	57.7%	16	48.5%	.282
Grade I–II (*n*, %)	23	44.2%	15	45.5%	.912
Grade III–IV (*n*, %)	7	13.5%	1	3.0%	.108
Incidence of chronic GVHD (any grade) (n, %)	3	5.8%	5	15.2%	.252
Cumulative incidence of acute or chronic GVHD (n, %)	31	59.6%	16	48.5%	.314
Immunological recovery in 360 days (*n*, %)	33/40 (82.5%)		13/18 (72.2%)		.371

Abbreviations: GVHD, Graft‐versus‐host disease; Haplo‐HSCT, haploidentical hematopoietic stem cell transplantation; MUD‐HSCT, matched unrelated donor hematopoietic stem cell transplantation; SD, standard deviation.

### 3.3 Outcome comparisons after transplant

We performed a comparison of the occurrences of acute and chronic GVHD between these two groups of patients. The data revealed the incidences of acute GVHD in patients undergoing haplo‐HSCT and MUD‐HSCT were 57.7% and 48.5%, respectively (*p* = .282). Both the haplo‐HSCT and MUD‐HSCT groups exhibited similar chances of grade I–II acute GVHD (44.2% vs. 45.5%; *p* = .282) and grade III–IV acute GVHD (13.5% vs. 3.0%; *p* = .108). Moreover, the incidences of chronic GVHD did not show substantial difference between haplo‐HSCT group and MUD‐HSCT group (5.8% vs. 15.2%; *p* = .252; Table 2).

Regarding the immunological recovery rate 1 year after allo‐HSCT, patients who underwent haplo‐HSCT had a likelihood of immunological recovery comparable to that in patients who underwent MUD‐HSCT (82.5% and 72.2%, respectively; *p* = .371; Table 2).

### 3.4 Survival comparisons

**FIGURE 1:**
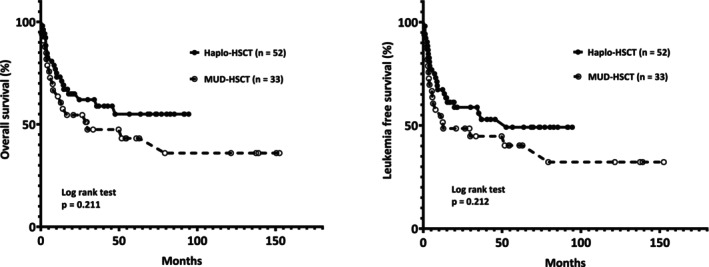
Overall survival and leukemia‐free survival comparisons. (A) The median overall survival period is not reached in the haploidentical hematopoietic stem cell transplant (haplo‐HSCT) group, while it is 29.8 months in the matched unrelated donor‐HSCT (MUD‐HSCT) group (*p* = .211). (B) The median leukemia‐free survival periods in haplo‐HSCT and MUD groups are 52.6 and 12.7 months, respectively (*p* = .212).

The median OS in patients receiving haplo‐HSCT was not reached, whereas it reached 29.8 months in patients undergoing MUD‐HSCT (*p* = .211; Figure 1A). Further analysis at specific time points showed that the 90‐day survival rates (94.2% vs. 87.9%; *p* = .298) and the 1‐year survival rates (73.1% vs. 63.6%; *p* = .339) did not exhibit substantial differences between these two groups (Table 2).

Median LFS durations for the haplo‐HSCT and MUD‐HSCT groups were 52.6 and 12.7 months (*p* = .212; Figure 1B). At the 90‐day mark, the LFS rates in both groups were 88.5% and 78.8% (*p* = .236). Moreover, 1‐year LFS rates in these two groups were 67.3% and 54.5%, respectively (*p* = .212; Table 2).

### 3.5 Prognostic factors

The univariate analysis indicated that haplo‐HSCT was not significantly correlated with an inferior LFS (HR, 0.69; 95% CI, 0.38–1.25; *p* = .216) or OS (HR, 0.67; 95% CI, 0.36–1.26; *p* = 0.214) compared to MUD‐HSCT (Table 3).

**TABLE 3:** Prognostic factors.

Clinical variables	Leukemia‐free survival						Overall survival					
	Univariate			Multivariate			Univariate			Multivariate		
	HR	95% CI	*p*	HR	95% CI	*p*	HR	95% CI	*p*	HR	95% CI	*p*
Age (years)	1.00	0.98–1.03	.688				1.01	0.99–1.04	.231			
Initial disease risk (high risk vs. non‐high risk)	1.11	0.60–2.12	.720				1.06	0.55–2.02	.869			
Charlson comorbidity Index (per score)	1.01	0.79–1.29	.949				1.05	0.83–1.34	.676			
EBMT risk score (per score)	1.41	1.10–1.82	.008	1.25	0.94–1.67	.128	1.44	1.10–1.87	.007	1.30	0.96–1.75	.091
Disease risk index (per score)	1.46	0.95–2.23	.082				1.26	0.81–1.95	.301			
Status before HSCT (non‐CR vs. CR)	2.81	1.38–5.72	.004	2.14	0.96–4.76	.062	2.48	1.17–5.23	.017	1.78	0.76–4.17	.186
Conditioning (non‐myeloablative vs. myeloablative)	0.95	0.51–1.76	.875				0.83	0.44–1.56	.559			
Time from diagnosis to HSCT (months)	1.00	1.00–1.00	.705				1.00	1.00–1.00	.606			
HSCT type (haplo‐HSCT vs. MUD‐HSCT)	0.69	0.38–1.25	.216				0.67	0.36–1.26	.214			
Donor age (years)	1.01	0.98–1.04	.472				1.01	0.99–1.04	.387			
Donor and recipient sex (different vs. same)	0.73	0.40–1.33	.309				0.65	0.35–1.22	.177			
ABO incompatibility (different vs. same)	0.58	0.32–1.05	.740				0.73	0.39–1.37	.329			
Acute GVHD (present/absent)	0.53	0.29–0.96	.035	0.56	0.31–1.02	.058	0.53	0.28–0.99	.048	0.56	0.30–1.06	.077
Chronic GVHD (present/absent)	0.88	0.31–2.46	.804				1.03	0.36–2.90	.956			

Abbreviations: CI, confidence interval; CR, complete remission; EBMT, European Group for Blood and Marrow Transplantation; GVHD, graft‐versus‐host disease; Haplo‐HSCT, haploidentical hematopoietic stem cell transplantation; HR, hazard ratio; MUD‐HSCT, matched unrelated donor hematopoietic stem cell transplantation.

We also explored other clinical variables that could potentially correlate with poorer LFS or OS. Univariate analysis identified a high EBMT risk score (HR, 1.41; 95% CI, 1.10–1.82; *p* = .008) and non‐CR status (HR, 2.81; 95% CI, 1.38–5.72; *p* = .004) as prognostic factors for diminished LFS. However, these associations did not attain statistical significance (*p* = .128 for high EBMT risk score; *p* = .062 for non‐CR status prior to allo‐HSCT) in multivariate analyses (Table 3).

In terms of OS, a high EBMT risk score (HR, 1.44; 95% CI, 1.10–1.87; *p* = .007) and non‐CR status (HR, 2.48; 95% CI, 1.17–5.23; *p* = .017) were found to be substantially associated with poorer OS by univariate analyses. However, these associations did not reach statistical significance in multivariate analyses (*p* = .091 for high EBMT risk score; *p* = .186 for non‐CR status prior to allo‐HSCT; Table 3).

## 4 DISCUSSION

The present study unveiled that individuals with acute leukemia undergoing haplo‐HSCT and MUD‐HSCT exhibited comparable rates of OS and LFS. Nonetheless, patients receiving haplo‐HSCT experienced a notably extended hospitalization period in contrast to their MUD‐HSCT counterparts. Additionally, it was observed that a high EBMT risk score and non‐CR status were substantially correlated with inferior OS and LFS.

Allo‐HSCT represents a possibly curative treatment choice for acute leukemia. Nonetheless, the task of identifying compatible donors is growing progressively more complex. Presently, HLA‐MSDs are accessible in only 30% of patients requiring Allo‐HSCT.^5^ Furthermore, likelihood of locating a MUD varies significantly among different ethnicities and across various nations. Replacing MUD‐HSCT with haplo‐HSCT presents ongoing challenges. One of the primary concerns related to haplo‐HSCT is the elevated probability of GVHD compared to the MUD‐HSCT. Patients undergoing haplo‐HSCT with a GVHD prophylaxis protocol containing PTCY, cyclosporine, and mycophenolate mofetil may encounter elevated rates of grade II–IV acute GVHD and moderate‐to‐severe chronic GVHD compared to individuals undergoing HSCT with allografts from HLA‐matched donors. Despite this, age over 60 years remains the sole independent factor predicting GVHD.^23^ Consistent with this result, our data also demonstrated that the rates of acute GVHD (57.7% vs. 48.5%; *p* = .282) and chronic GVHD (5.8% vs. 15.2%; *p* = .252) did not exhibit substantial differences between individuals receiving haplo‐HSCT and MUD‐HSCT. This suggests that a PTCY‐based triple‐combination GVHD prophylaxis regimen partially addresses the concerns regarding the occurrence of severe GVHD in haplo‐HSCT recipients. A study conducted by Bolaños‐Meade et al.^24^ further supported this hypothesis, showing that in patients undergoing allogeneic HLA‐matched HSCT with reduced‐intensity conditioning, the one‐year GVHD‐free rate was significantly higher in patients treated with a regimen of PTCY, tacrolimus, and mycophenolate mofetil compared to those receiving tacrolimus and methotrexate.

Notably, extremely profound immunosuppression for GVHD prophylaxis can increase the chance of CMV reactivation, particularly in regions with a high CMV prevalence.^16^ Furthermore, research has suggested that the occurrence of CMV reactivation tends to be higher in haplo‐HSCT recipients than in those receiving MUD‐HSCT.^6^ Addressing this challenge, CMV prophylaxis using letermovir^11^ or low‐dose valganciclovir^10^ has considerably diminished the likelihood of CMV reactivation in haplo‐HSCT recipients. These prophylactic measures have further contributed to overcoming the obstacles of replacing MUD‐HSCT with haplo‐HSCT. In the present analysis, we did not directly compare CMV reactivation incidences between these two groups of patients because of evolving nature of CMV prophylactic strategies implemented at our institution. Nonetheless, among acute leukemia individuals who received allo‐HSCT while receiving low‐dose valganciclovir prophylaxis, the cumulative rate of CMV DNAemia at week 14 notably diminished to just 15.0%. This underscores a substantial 88% reduction in the risk of CMV DNAemia at week 14 due to prophylactic lower‐dose valganciclovir.^10^

Importantly, the present study establishes that the haplo‐HSCT group and MUD‐HSCT group demonstrated comparable median OS durations (not reached vs. 29.8 months; *p* = .211) and LFS periods (52.6 vs. 12.7 months; *p* = .212). These outcomes were further corroborated by the logistic regression model, which affirmed that haplo‐HSCT carried no higher risk for inferior LFS (HR, 0.69; 95% CI, 0.38–1.25; *p* = .216) or OS (HR, 0.67; 95% CI, 0.36–1.26; *p* = .214) in comparison to MUD‐HSCT. These results are reinforced by the discoveries made by Solomon et al.,^25^ which similarly conclude that haplo‐HSCT or MUD‐HSCT did not exert substantial influence on the outcomes of individuals who received allo‐HSCT for their acute leukemia. Specifically, both haplo‐HSCT and MUD‐HSCT are considered valid options for ≥60‐year‐old AML patients. These two approaches have been shown to yield comparable OS and LFS.^26^

The current study revealed that a high EBMT risk score and non‐CR status, rather than donor type, were significantly correlated with worse OS and LFS. Significantly, the EBMT score exhibited robust predictive capacity for OS and proved to be a valuable tool for categorizing the threat of acute leukemia relapse in recipients of allo‐HSCT.^27^ Additionally, the existence of non‐CR status before undergoing allo‐HSCT consistently correlated with a heightened risk of acute leukemia relapse.^28^ These findings support and validate the results of our study.

The retrospective nature and relatively small study cohort were the major limitations in the present study. Additionally, the inclusion of a wide range of time may have introduced bias. Additionally, molecular genetic profiles were unavailable for some of the patients in earlier periods. To address these limitations, further prospective and randomized control studies, which are necessary to determine whether haplo‐HSCT can conditionally replace MUD‐HSCT as a treatment option for acute leukemia, should be conducted.

In conclusion, our study demonstrated no substantial differences in OS or LFS between patients with acute leukemia receiving haplo‐HSCT and MUD‐HSCT. Moreover, our data identified both a high EBMT risk score and non‐CR status as factors significantly linked to poorer OS and LFS in acute leukemia patients receiving allo‐HSCT. Haplo‐HSCT may serve as an alternative to MUD‐HSCT for the treatment of acute leukemia, offering similar survival outcomes.

## AUTHOR CONTRIBUTIONS

Yin‐Che Wang: Conceptualization, investigation, formal analysis. Cheng‐Lun Lai: Conceptualization, Methodology, Investigation, Writing – review & editing. Tsung‐Chih Chen: Methodology, Investigation. Jyh‐Pyng Gau: Conceptualization, Writing – review & editing. Chieh‐Lin Jerry Teng: Conceptualization, Supervision, Writing – review & editing.

## CONFLICT OF INTEREST STATEMENT

Chieh‐Lin Jerry Teng received honorarium and consulting fees from Novartis, Roche, Pfizer, Takeda, Johnson and Johnson, Amgen, BMS Celgene, Kirin, and MSD. The other authors have no conflicts of interest.

## ETHICS STATEMENT

Participant details were de‐identified, and the study was conducted according to the current version of the Declaration of Helsinki. The Institutional Review Board of Taichung Veterans General Hospital approved the study (CE23259C).

## Data Availability

The datasets used and/or analyzed during the current study are available from the corresponding author on reasonable request.
